# Health-related quality of life and psychosocial outcomes in long-term survivors treated with immune checkpoint inhibitors: a nationwide multicenter study

**DOI:** 10.3389/fimmu.2025.1693295

**Published:** 2025-12-10

**Authors:** Taha Koray Sahin, Fatih Atalah, Bahadir Koylu, Ahmet Oruc, Aydin Acarbay, Akgun Karakok, Sevgi Dogan, Cevat Ilteris Kikili, Görkem Turhan, Gamze Emin, Selahattin Celik, Ilknur Deliktas Onur, Tuba Ugur Tuzcu, Sedat Biter, Safa Can Efil, Fadime Sinem Ardic, Azer Gokmen, Halil Goksel Guzel, Kadri Turan, Ahmet Kürsad Disli, Salih Tunbekici, Erdem Goker, Mevlude Inanc, Ozlem Ozdemir, Banu Ozturk, Muslih Urun, Hatime Arzu Yasar, Mehmet Ali Nahit Sendur, Ismail Oguz Kara, Osman Sutcuoglu, Ozturk Ates, Tulay Eren, Atila Yildirim, Saadettin Kilickap, Omer Dizdar, Fatma Alev Turker, Mustafa Erman, Mehmet Artac, Fatih Selcukbiricik, Sercan Aksoy, Deniz Can Guven

**Affiliations:** 1 Department of Medical Oncology, Hacettepe University Cancer Institute, Ankara, Türkiye; 2 Department of Medical Oncology, Faculty of Medicine, Medeniyet University, Prof. Dr. Süleyman Yalçın City Hospital, Istanbul, Türkiye; 3 Department of Medical Oncology, Koç University School of Medicine, Istanbul, Türkiye; 4 Department of Medical Oncology, Necmettin Erbakan University School of Medicine, Konya, Türkiye; 5 Department of Medical Oncology, Karadeniz Technical University, Trabzon, Türkiye; 6 Department of Medical Oncology, Etlik City Hospital, Ankara, Türkiye; 7 Department of Medical Oncology, Health Sciences University Dr Abdurrahman Yurtaslan Ankara Oncology Education and Research Hospital, Ankara, Türkiye; 8 Department of Medical Oncology, Gazi University School of Medicine, Ankara, Türkiye; 9 Department of Medical Oncology, Faculty of Medicine, Çukurova University, Adana, Türkiye; 10 Department of Medical Oncology, Bilkent City Hospital, University of Health Sciences, Ankara, Türkiye; 11 Department of Medical Oncology, Ankara University Faculty of Medicine, Ankara, Türkiye; 12 Department of Medical Oncology, Van Yüzüncü Yıl University Medical Faculty, Van, Türkiye; 13 Department of Medical Oncology, Antalya Training and Research Hospital, Antalya, Türkiye; 14 Department of Medical Oncology, Izmir City Hospital, Izmir, Türkiye; 15 Department of Medical Oncology, Erciyes University, Kayseri, Türkiye; 16 Department of Medical Oncology, Ege University, Izmir, Türkiye; 17 Department of Medical Oncology, Istinye University Faculty of Medicine, Istanbul, Türkiye

**Keywords:** immune checkpoint inhibitors, long-term survivors, quality of life, fear of progression, survivorship care

## Abstract

**Background:**

The increasing use of immune checkpoint inhibitors (ICIs) has resulted in a growing population of long-term survivors (LTS). However, the long-term psychosocial and quality of life (QoL) outcomes in these patients remain underexplored. This study aimed to evaluate QoL, psychological morbidity, fear of cancer progression (FoP), and functional outcomes in cancer patients treated with ICIs for at least six months without disease progression.

**Methods:**

This cross-sectional, multicenter study included 346 adult cancer patients from 17 tertiary oncology centers in Türkiye. Participants had received ICIs for ≥6 months in (neo)adjuvant or metastatic settings. Standardized questionnaires assessed QoL, psychological distress, FoP, immune-related adverse events (irAEs), and work status.

**Results:**

The median age of the cohort was 62 years (IQR: 53–69). Average survivor QoL was comparable to the Turkish general population; but 119 (34.5%) survivors had poor QoL. Clinically relevant symptoms of depression and anxiety were present in 24.3% and 20.8% of patients, respectively, while 48% reported FoP. The overall return-to-work rate among patients initially employed was 50.9%, with 72.7% returning within the first year. Depression, anxiety, and FoP were negatively correlated with all QoL domains. All grade irAEs were common (53.8%) but not significantly associated with worse QoL (p=0.149).

**Conclusions:**

This study represents one of the largest cohorts to date evaluating survivorship issues among LTS treated with ICIs. Among patients receiving ICIs for at least six months, nearly one-third experienced impaired QoL, primarily driven by psychological distress and FoP. Further research is needed to address survivorship care in this population.

## 1 Introduction

The advent of immune checkpoint inhibitors (ICIs) has revolutionized the therapeutic landscape of many cancers, particularly those historically associated with poor prognosis, such as melanoma, renal cell carcinoma (RCC), and non-small cell lung cancer (NSCLC) (1). By targeting immune checkpoints, including cytotoxic T-lymphocyte-associated protein 4 (CTLA-4) and programmed death-1 (PD-1)/PD-ligand 1 (PD-L1), ICIs have elicited durable responses and survival gains across multiple tumor types (2–4). Long-term data from pivotal trials such as CheckMate 067 have revealed that the median overall survival (OS) with nivolumab plus ipilimumab has reached nearly six years, with 10-year survival rates exceeding 40% in advanced melanoma, marking a remarkable shift from the pre-ICI era, where median OS was often less than one year (5). These long-term benefits have contributed to the emergence of a growing population of long-term survivors (LTS) who have either completed or continue ICI treatment. Moreover, ICIs have moved to adjuvant and neoadjuvant settings, further expanding the group of patients exposed to this class of agents (6–8).

With the substantial improvement in survival achieved through ICIs, increasing attention has shifted towards the survivorship phase of cancer care. Survivorship refers not only to cancer control but also to the enduring physical, psychosocial, and functional challenges faced by LTS (9). Furthermore, ICIs can lead to class-specific chronic immune-related adverse events (irAEs) involving the endocrine, dermatologic, pulmonary, and gastrointestinal systems, some of which, such as hypophysitis and thyroiditis may require lifelong hormone replacement (10, 11). Although clinical trials often report stable quality of life (QoL) during ICI therapy, real-world survivors frequently experience persistent fatigue, neurocognitive symptoms, and functional impairments that are not captured in trial settings (12–15). Psychosocial distress, particularly anxiety, depression, and fear of progression (FoP), is prevalent among survivors of advanced cancer and may significantly affect QoL (16, 17). Additionally, domains such as return to work (RTW) remain underexplored in ICI survivorship. Despite these multifaceted challenges, most available data on survivorship originate from small, single center studies from patients treated within the earlier lines and in high resource settings leaving substantial knowledge gaps (18, 19). The survivorship issues could be more pronounced in the patients treated in the later lines, in the patients with more comorbidities treated in the real life settings instead of the clinical trials and patients treated in the low and middle-income countries (LMIC) (20, 21).

To address these gaps, we conducted a nationwide, multicenter study evaluating health-related QoL, psychological morbidity, and functional outcomes in LTS treated with ICIs across Türkiye. Additionally, by comparing our findings with normative Turkish population data, we aimed to contextualize survivorship outcomes within the setting of a lower-middle-income country.

## 2 Materials and methods

### 2.1 Study design and setting

This was a cross-sectional, observational study conducted across 17 academic and tertiary oncology centers in Türkiye between January 2, 2025, and April 30, 2025. Patients were eligible for inclusion in this study if they were aged 18 years or older, had a histologically confirmed diagnosis of a malignant solid tumor, and had been receiving ICIs for at least six months without radiological or clinical evidence of disease progression at the time of study inclusion. Both patients treated in the metastatic setting and those receiving ICIs as part of (neo)adjuvant therapy for high-risk early-stage disease were included. All participants provided written informed consent prior to study participation. Patients were excluded if they had evidence of progressive disease, received ICIs for less than six months, or had severe cognitive or psychiatric impairment that precluded informed consent or valid questionnaire completion.

### 2.2 Data collection

At the study visit, data were collected using a structured, self-administered questionnaire during in-person visits. Demographic and clinical data were obtained from medical records and patient self-reports, including age, sex, marital status, education level, cancer diagnosis and stage, current work status, RTW, Eastern Cooperative Oncology Group (ECOG) performance status at the time of survey, details of ICI treatment (type of ICI, mono- *vs*. combination therapy, line of therapy, and duration of treatment), and the occurrence of any irAEs during ICI therapy. irAEs were defined as any grade immune-mediated toxicity as documented by treating physicians, using Common Terminology Criteria for Adverse Events (CTCAE) v5.0 criteria (22). For irAEs, we recorded the involved organ system (endocrine, dermatologic, gastrointestinal, pulmonary, neurological, etc.), the maximum grade, the use of systemic corticosteroids for management, and presence of permanent ICI discontinuation due to toxicity.

### 2.3 Measures

#### 2.3.1 Quality of life

The European Organization for Research and Treatment of Cancer Quality of Life Questionnaire-Core 30 (EORTC QLQ-C30) was used to evaluate general cancer-specific QoL. This instrument includes a Global Health Status/QoL scale and five functional subscales (Physical, Role, Emotional, Cognitive, and Social functioning), as well as symptom scales. Scores are linearly transformed to 0–100 scales, with higher scores on functional scales and global health indicating better QoL, and higher symptom scores indicating greater symptom burden. Poor QoL was defined as a Global Health Status score on the EORTC QLQ-C30 that was ≥10 points below the group mean (23–25). We obtained Turkish general population normative means for QLQ-C30 scales from published literature (26).

#### 2.3.2 Psychological distress

Symptoms of anxiety and depression were measured with the Hospital Anxiety and Depression Scale (HADS). HADS is a 14-item questionnaire yielding two subscales: Anxiety (HADS-A) and Depression (HADS-D), each ranging 0–21. We used the conventional cutoff of ≥8 on each subscale to indicate the presence of at least mild clinically relevant anxiety or depression symptoms. We also recorded HADS total score (sum of A+D, range 0–42) for an overall distress measure. Patients with HADS-A or HADS-D ≥8 were classified as having anxiety or depression, respectively, in analyses (27).

#### 2.3.3 Fear of cancer progression

We assessed fear of cancer progression using the Fear of Cancer Recurrence Inventory-Short Form (FCRI-SF), adapted in wording to refer to progression since many patients had not had a complete remission. The FCRI-SF is a brief validated instrument measuring the severity of fear of cancer recurrence/progression (28). It yields a total score (range 0–36, higher = greater fear). A score of 13 or above on the FCRI-SF has been suggested as a cutoff for at least moderate levels of fear of progression (FoP) requiring attention (29, 30).

### 2.4 Statistical analysis

Descriptive statistics were used to summarize patient characteristics and outcome measures. Continuous variables are presented as mean with standard deviation (SD) or median with interquartile range (IQR), as appropriate. Categorical variables are presented as counts and percentages. We conducted univariate analyses to identify factors associated with poor QoL, using chi-square tests for categorical factors and t-tests or Mann-Whitney tests for continuous factors, as appropriate. Variables of interest included age (<65 *vs* ≥65), sex, marital status, education level, ECOG performance status (dichotomized as 0 *vs* ≥1), treatment setting (metastatic *vs* adjuvant), duration of ICI therapy, combination *vs* monotherapy, presence of any irAE (yes *vs* no), active work status before the start of ICIs (yes *vs* no), and presence of psychological distress (depression/anxiety yes *vs* no) or FoP (yes *vs* no). We then constructed a multivariable logistic regression model to determine independent predictors of poor QoL (yes/no). All variables with p<0.10 in univariate screening were considered for inclusion in the multivariate model, and a stepwise backward elimination was applied to retain factors with p<0.05. Adjusted odds ratios (aOR) with 95% confidence intervals (CI) are reported for the final model. We also examined Pearson’s or Spearman’s correlations between continuous all QoL domain scores and continuous psychological scores (HADS, FCRI). All statistical analyses were performed in SPSS, version 24.0 (IBM Inc., Armonk, NY, USA), and p-values below 0.05 were considered the threshold limit for statistical significance.

## 3 Results

### 3.1 Patient characteristics

A total of 346 LTS receiving ICIs were included across the 17 centers. Baseline characteristics of the study cohort are summarized in **Table 1**. The median age at the time of survey was 62 years (IQR: 53–69), and 43.4% of LTS were aged 65 or older. 67.1% of the LTS were male (232/346) and about 59% had a high school education or less. Overall, 89.6% of LTS had an ECOG performance status of 0–1 (41.9% ECOG 0 and 47.7% ECOG 1) and only 10.4% had ECOG 2.

**Table 1:** Baseline patient characteristics of study cohort (n=346).

Characteristics	n, (%)
Age at ICI treatment, median (IQR)	62 (53-69)
Age	
<65 years	196 (56.6)
≥65 years	150 (43.4)
Sex	
Female	114 (32.9)
Male	232 (67.1)
Marital Status	
Single or divorced	61 (17.6)
Married	285 (82.4)
Education	
High school or below	203 (58.7)
Higher than high school	143 (41.3)
ECOG performance status, n (%)	
0	145 (41.9)
1	165 (47.7)
2	36 (10.4)
Primary Tumor, n (%)	
NSCLC	163 (47.1)
RCC	63 (18.2)
Melanoma	47 (13.6)
GI cancer	18 (5.2)
Breast	13 (3.8)
Others	42 (12.1)
Number of ICI Lines, n (%)	
1	154 (44.5)
2	158 (45.7)
3 or more	34 (9.8)
Treatment Setting	
Neoadjuvant and/or Adjuvant	39 (11.3)
Metastatic	307 (88.7)
Type of ICI	
Nivolumab	215 (62.1)
Pembrolizumab	83 (24.0)
Atezolizumab	22 (6.4)
Nivolumab-Ipilimumab	15 (4.3)
Durvalumab	7 (2)
Avelumab	4 (1.2)
Months of ICI treatment	
6-12	136 (39.3)
12-24	107 (30.9)
24-36	48 (13.9)
>36	55 (15.9)
Type of treatment, *n* (%)	
Monotherapy	255 (73.7)
Combination therapy	91 (26.3)

ICI, Immune Checkpoint Inhibitor; IQR, Interquartile Range; ECOG, Eastern Cooperative Oncology Group; NSCLC, Non-Small Cell Lung Cancer; RCC, Renal Cell Carcinoma; GI, Gastrointestinal.

NSCLC was the most common primary cancer (47.1%), followed by RCC (18.2%), and melanoma (13.6%). The majority of LTS(88.7%) were treated in the metastatic setting, while 11.3% received ICIs as adjuvant or neoadjuvant therapy. At the time of survey, 60% had received ICI therapy for over one year, and 29.8% for more than two years. Most LTS received ICI monotherapy (74%), with nivolumab (62.1%) and pembrolizumab (24.0%) being the most frequently used agents. Over half of the LTS (55.5%) were treated with ICIs in second or later lines.

### 3.2 Immune-related adverse events

Among all LTS, 53.8% (n=186) experienced at least one irAE of any grade during their ICI therapy (**Table 2**). The rate of irAEs was highest in the nivolumab-ipilimumab subgroup (86.7%), followed by atezolizumab (63.6%) and pembrolizumab (51.8%). Endocrine irAEs (27.7%) and cutaneous irAEs (22.5%) were the most frequently reported irAEs. Gastrointestinal (8.1%), pulmonary (3.5%), and neurologic (0.6%) irAEs were less commonly observed.

**Table 2:** Immune-related adverse events associated by type of ICI (n=346).

	Nivolumab (n=215)	Pembrolizumab (n=83)	Atezolizumab (n=22)	Nivolumab-Ipilimumab (n=15)	Durvalumab (n=7)	Avelumab (n=4)
Any irAEs, n(%)	111 (51.6)	43 (51.8)	14 (63.6)	13 (86.7)	4 (57.1)	2 (50)
Endocrine, n(%)	58 (27)	24 (28.9)	8 (36.4)	6 (40)	2 (28.6)	1 (25)
Cutaneous, n(%)	45 (20.9)	21 (25.3)	9 (40.9)	8 (53.3)	1 (14.3)	1 (25)
Gastrointestinal, n(%)	15 (7)	8 (9.6)	1 (4.5)	4 (26.7)	1 (14.3)	0 (0)
Pulmonary, n(%)	8 (3.7)	1 (1.2)	1 (4.5)	2 (13.3)	0 (0)	0 (0)
Neurologic, n(%)	1 (0.5)	0 (0)	0 (0)	1 (6.7)	0 (0)	0 (0)
Grade 3 or higher iRAE, n(%)	11 (5.1)	8 (9.6)	2 (9.1)	6 (40)	1 (14.3)	0 (0)
Systemic corticosteroid, *n* (%)	23 (10.7)	8 (9.6)	2 (9.1)	7 (46.7)	1 (14.3)	0 (0)
Discontinuation of ICI, n (%)	13 (6)	8 (9.6)	2 (9.1)	4 (26.7)	1 (14.3)	1 (25)

ICI, Immune Checkpoint Inhibitor; irAEs, Immune-Related Adverse Events.

Grade 3 or higher irAEs occurred in 8.4% of patients, with the highest incidence (40%) observed among those treated with combination nivolumab-ipilimumab therapy (6/15). Systemic corticosteroids were required in 12.2% of patients, and 8.4% permanently discontinued ICI due to toxicities.

### 3.3 Quality of life, psychological morbidity, and functional outcomes

**Figure 1:**
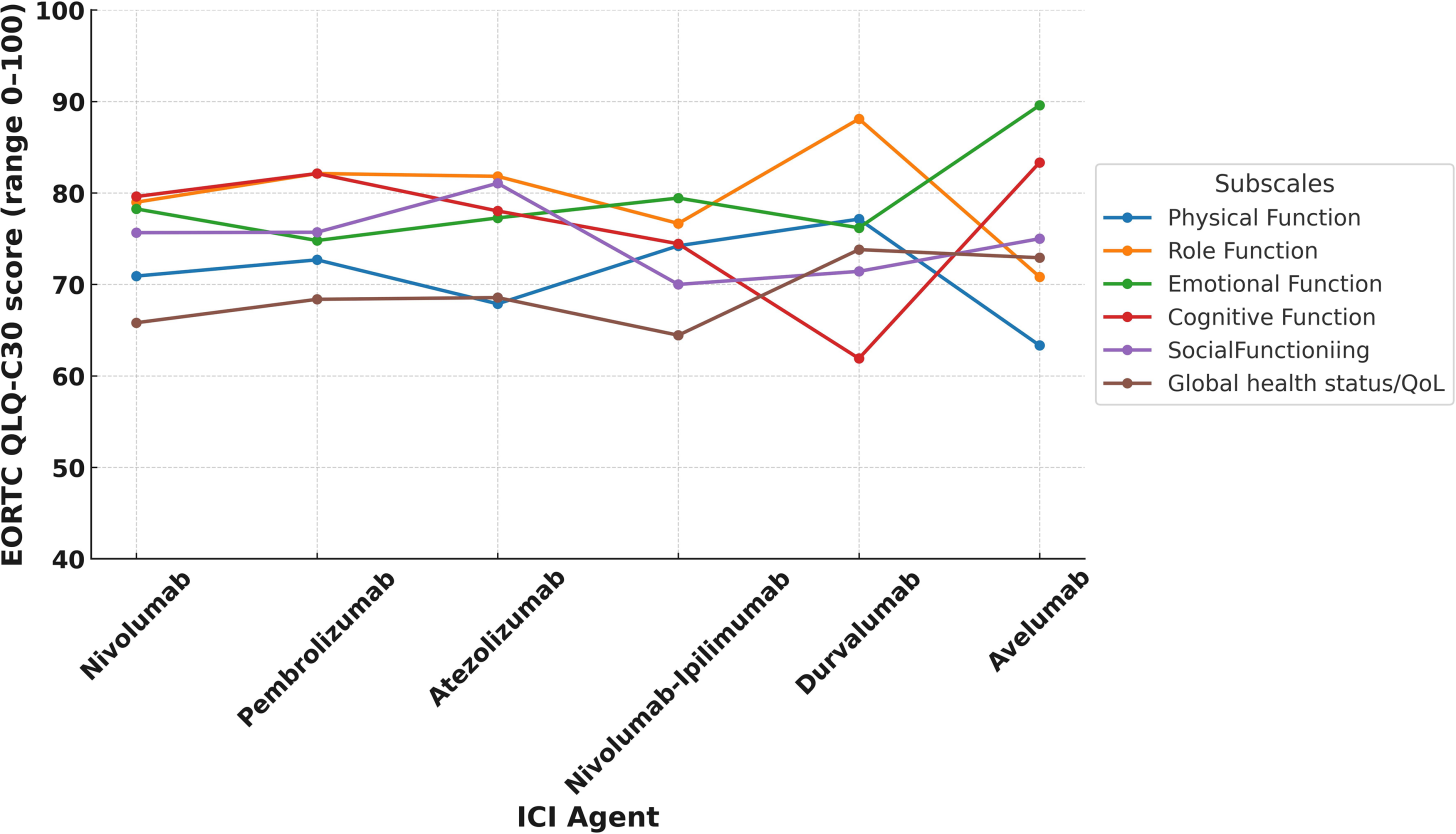
EORTC QLQ-C30 functional subscale score by ICI agent.

**Figure 2:**
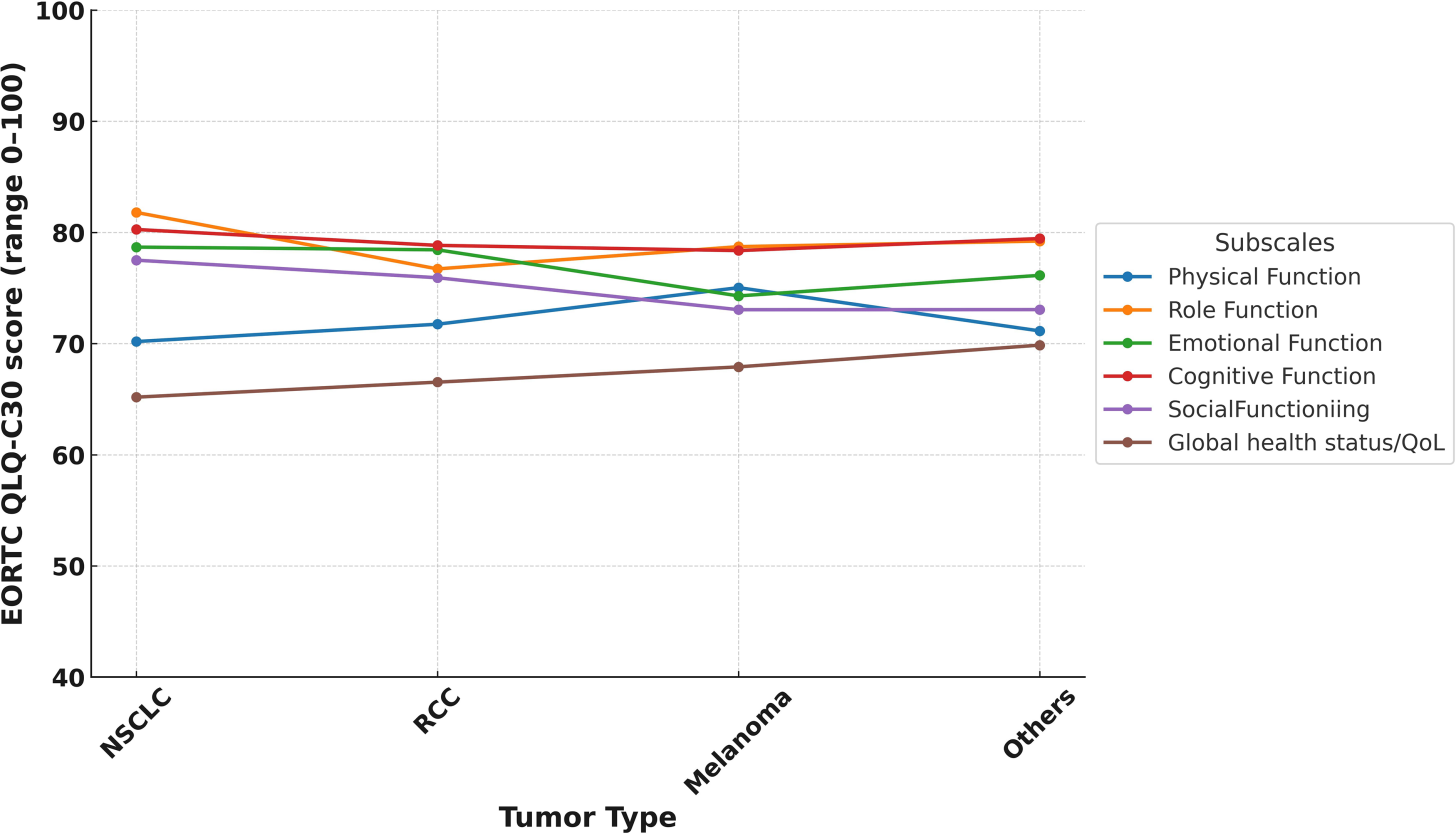
EORTC QLQ-C30 functional subscale score by tumor type.

**Figure 3:**
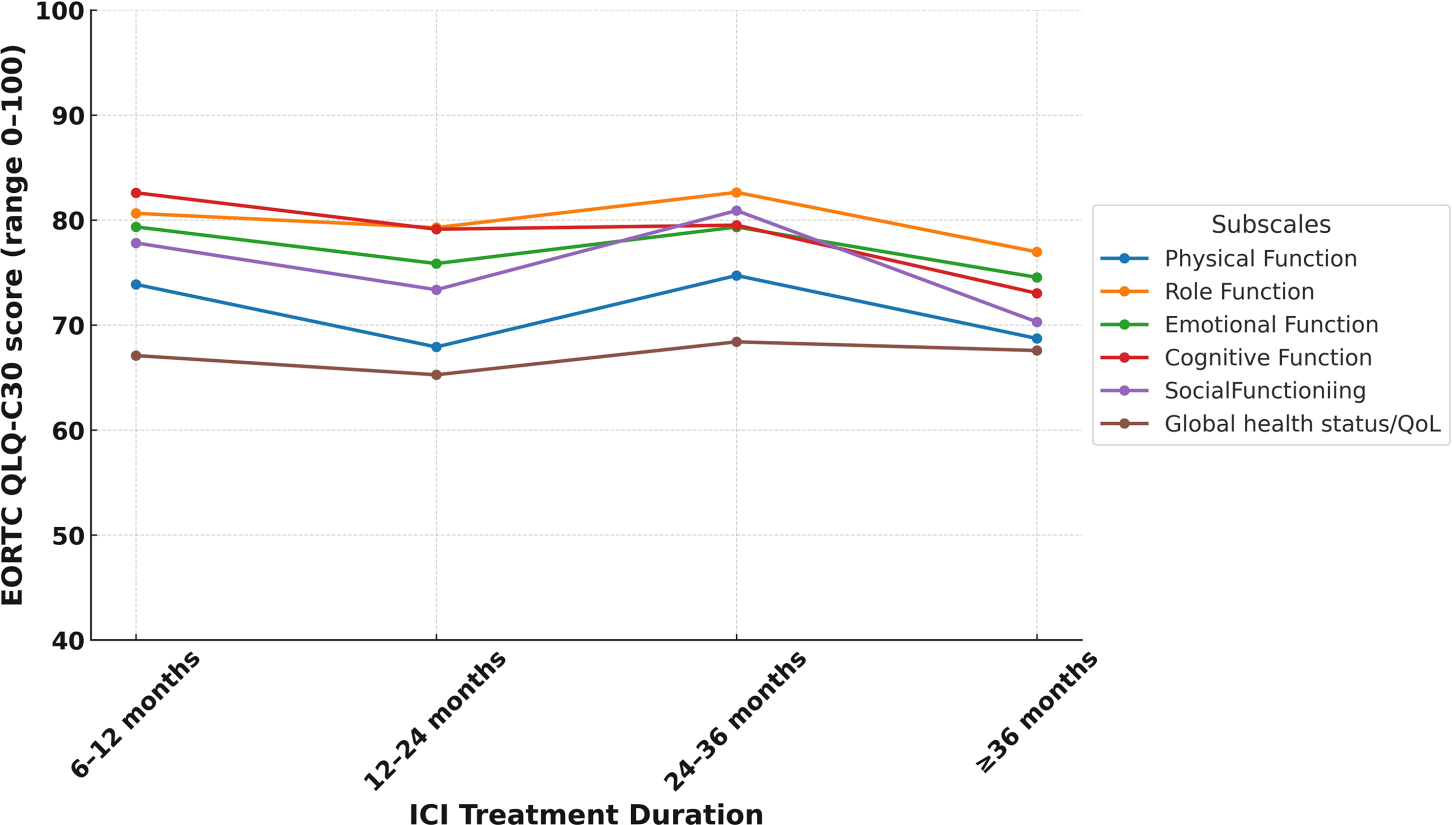
EORTC QLQ-C30 functional subscale score by duration of ICI treatment.

In the study cohort, the mean global health status score was 66.8 (SD: 21.9), which was modestly higher than the Turkish general population reference value of 60.7 (SD: 19.8) (**Table 3**). **Supplementary Table 1** illustrates the distribution of scores, revealing that about 15.6% of LTS gave very high global health scores rating (91–100). Despite these positive averages, 34.5% (n=119) of LTS were categorized as having poor QoL. Functional domain scores were generally comparable to reference values. The highest mean score was observed in role functioning (mean = 79.9, SD = 23.6), followed by cognitive functioning (mean = 79.6, SD = 21.3) and emotional functioning (mean = 77.5, SD = 20.2). Physical functioning had the lowest functional score at 71.3 (SD = 21.7). Functional domain scores varied across ICI agents, with relatively higher global health and physical functioning observed among patients treated with durvalumab and pembrolizumab compared to those receiving nivolumab-ipilimumab (**Figure 1**). Similarly, differences by tumor type were noted, with LTS with RCC reporting slightly better scores than those with NSCLC or melanoma (**Figure 2**). Although no consistent pattern was seen across ICI treatment durations, patients treated for 24–36 months demonstrated modestly better physical functioning (**Figure 3**).

**Table 3:** Patient quality of life, fear of progression, psychological and work-related outcomes.

Domain	Measure	Total (n=346)	Turkish population
		Mean (SD)	Mean (SD)
Quality of Life (EORTC QLQ-C30)	Global health status	66.8 (21.9)	60.7 (19.8)
	Physical functioning	71.3 (21.6)	75.8 (16.7)
	Role functioning	79.9 (23.5)	82.3 (22.4)
	Emotional functioning	77.5 (20.2)	65.8 (25.5)
	Cognitive functioning	79.5 (21.3)	75.5 (23.2)
	Social functioning	75.7 (24.5)	83.1 (23.6)
Psychological Issues (HADS)	HADS total score	9.73 (7.6)	N/A
	At least mild anxiety (≥8)	72 (20.8%)	
	At least mild depression (≥8)	84 (24.3%)	
Fear of Progression (FCRI-SF)	FCRI-SF total score	12.72 (7.9)	N/A
	At least mild FoP (≥13)	166 (48%)	
Work Status	Working at the start of ICI	173 (50%)	N/A
	Working at time of study visit	88 (25.4%)	
	Return to work <6 months	33 (37.5%)	
	Return to work 6–12 months	31 (35.2%)	
	Return to work >12 months	24 (27.2%)	

EORTC QLQ-C30, European Organisation for Research and Treatment of Cancer Quality of Life Questionnaire–Core 30; HADS, Hospital Anxiety and Depression Scale; FCRI-SF, Fear of Cancer Recurrence Inventory-Short Form; FoP, Fear of Progression; SD, Standard Deviation; ICI, Immune Checkpoint Inhibitor.

Among the EORTC QLQ-C30 symptom scales, financial difficulties had the highest symptom burden with a mean score of 24.3 (SD = 28.6), followed by dyspnea at 23.5 (SD = 28.6) and pain at 21.9 (SD = 26.0). In contrast, vomiting and diarrhea were the least reported symptoms, with mean scores of 10.0 (SD = 18.8) and 7.9 (SD = 19.2), respectively.

Based on the HADS, 24.3% (n=84) of participants met the threshold for clinically meaningful depression (HADS-D ≥8), and 20.8% (n=72) met the threshold for anxiety (HADS-A ≥8). The mean HADS total score across the cohort was 9.73 (SD = 7.6). In addition, nearly half of the patients (48%, n=166) reported moderate-to-severe FoP, with a mean FCRI-SF score of 12.7 (SD = 7.9).

A significant inverse correlation was observed between psychological symptoms and all QoL subdomains (**Table 4**). The strongest correlation was between emotional functioning and anxiety (r = –0.616, p<0.001), followed by emotional functioning and depression (r = –0.524, p<0.001). Depression, anxiety, and FoP were also negatively correlated with global health status (r = –0.489, –0.404, and –0.348 respectively, all p<0.001).

**Table 4:** Correlations between QoL domains and depression, anxiety, and FoP.

	Depression		Anxiety		Fear of Progression	
QoL Domain	Correlation coefficient (r)	p value	Correlation coefficient (r)	p value	Correlation coefficient (r)	p value
Global health status	-0.489	<0.001	-0.404	<0.001	-0.348	<0.001
Physical functioning	-0.416	<0.001	-0.382	<0.001	-0.322	<0.001
Role functioning	-0.396	<0.001	-0.381	<0.001	-0.283	<0.001
Emotional functioning	-0.524	<0.001	-0.616	<0.001	-0.441	<0.001
Cognitive functioning	-0.444	<0.001	-0.456	<0.001	-0.312	<0.001
Social functioning	-0.482	<0.001	-0.470	<0.001	-0.356	<0.001

Regarding employment status, 50% of participants (n=173) were actively working at the time of ICI initiation, but only 25.4% (n=88) were still employed at the time of the survey. Among those who did return to work, 37.5% (n=33) did so within six months, 35.2% (n=31) within 6–12 months, and 27.2% (n=24) after one year or more.

### 3.4 Factors associated with poor quality of life

Univariate analysis identified several factors significantly associated with poor QoL: ECOG ≥1 (OR: 2.03, 95% CI: 1.28–3.22; p = 0.003), metastatic treatment setting (OR: 5.69, 95% CI: 1.97–16.42; p = 0.001), depression (OR: 5.59, 95% CI: 3.29–9.50; p< 0.001), anxiety (OR: 5.11, 95% CI: 2.93–8.92; p < 0.001), and FoP (OR: 3.67, 95% CI: 2.30–5.84; p< 0.001) (**Table 5**). In the multivariable logistic regression model, the following remained independent predictors of poor QoL: ECOG ≥1 (adjusted OR: 1.96, 95% CI: 1.14–3.38, p=0.015), metastatic setting (aOR: 3.62, 95% CI: 1.16–11.27, p = 0.027), presence of depression (aOR: 2.74, 95% CI: 1.44–5.21, p = 0.002), anxiety (aOR: 2.43, 95% CI: 1.20–4.92, p = 0.013), and presence of FoP (aOR: 2.03, 95% CI: 1.18–3.48, p=0.038). Other variables including age, sex, duration of ICI treatment, and the presence of irAEs were not significant predictors for poor QoL.

**Table 5:** Logistic regression analysis of poor quality of life in patients treated with ICIs (n=346).

Variables	Univariate			Multivariate		
	OR	95% CI	p-Value	aOR	95% CI	p-Value
Age (<65 years *vs* ≥65 years)	1.24	0.79-1.93	0.344			
Sex (female *vs* male)	1.07	0.67-1.70	0.416			
ECOG performance status (1–2 *vs* 0)	2.03	1.28-3.22	0.003	1.96	1.14-3.38	0.015
Education (Higher than high school *vs* High school or below)	0.68	0.41-1.12	0.133			
Treatment setting (metastatic *vs* (neo)adjuvant)	5.69	1.97-16.42	0.001	3.62	1.16-11.27	0.027
Received other systemic treatments with ICI (yes *vs* no)	0.88	0.53-1.46	0.634			
Presence of irAEs (yes *vs* no)	1.38	0.89-2.16	0.149			
Working at time of study visit (yes *vs* no)	0.67	0.40-1.13	0.138			
Months of ICI treatment (24 months ≥ *vs <*24 months	1.05	0.65-1.70	0.829			
Depression (yes *vs* no)	5.59	3.29-9.50	<0.001	2.74	1.44-5.21	0.002
Anxiety (yes *vs* no)	5.11	2.93-8.92	<0.001	2.43	1.20-4.92	0.013
Fear of progression (yes *vs* no)	3.67	2.30-5.84	<0.001	2.03	1.18-3.48	0.038

aOR, Adjusted Odds Ratio; CI, Confidence Interval; ECOG, Eastern Cooperative Oncology Group; ICI, Immune Checkpoint Inhibitor; irAEs, Immune-Related Adverse Events; OR, Odds Ratio.

## 4 Discussion

In this large multicenter cohort of LTS treated with ICIs, we observed that despite comparable average health-related quality of life (HRQoL) scores general population, approximately one-third of the ICI-treated LTS reported poor QoL despite having no evidence of disease progression. Depression and anxiety were present in over 20% of patients, and nearly half reported clinically relevant FoP. Although irAEs are frequent, there was no significant association with lower QoL, assuring the safety of ICIs on affecting QoL. Higher levels of depression, anxiety, and FoP were strongly correlated with worse QoL.

In our nationwide multicenter study, one-third of ICI-treated LTS reported poor QoL, despite mean global health scores being comparable to those of the general population. Preserved or even better global health status has previously been reported in cancer survivors of advanced cancer treated with ICIs (13, 31, 32). Similar to our results, in a single-center study from Memorial Sloan Kettering Cancer Center, Mamoor et al. reported that advanced melanoma survivors (n=90) exhibited generally favorable QoL, with 60% maintaining high global health scores a median of 40 months after initiating ICIs, although fatigue and joint pain were commonly reported symptoms (13). Similarly, although the sample size was small (n=90), Looman et al. found that despite persistent long-term physical symptoms such as fatigue, muscle and joint pain, most melanoma survivors rated their health and HRQoL positively on standardized measures (14). Candido et al. recently reported that 28% of ICI survivors (across melanoma, NSCLC, and RCC) had clinically poor QoL despite average global health scores comparable to reference norms (23). In contrast to these data and our cohort, in a real-world lung cancer cohort derived from a patient registry (n=226), Jim et al. observed that patients treated with ICIs had significantly lower functional and emotional QoL scores compared to general US population and normative cancer populations, with fatigue, aching joints, and muscle pain being the most frequent moderate-to-severe complaints (32). In evaluating the factors associated with poor QoL in these populations, prior studies have identified age (both younger and older age) and the need for additional therapy as relevant predictors (33, 34). We did not find a significant association between poor QoL and patient age or line of the treatment, possibly due to differences in population characteristics, study design, and timing of assessments.

Psychosocial morbidity emerged as a significant and multifaceted issue in our cohort, with approximately one-quarter of LTS screened positive for depression (24.3%) or anxiety (20.8%). These rates surpass those reported in a large meta-analysis of long-term cancer survivors, which identified pooled prevalence estimates of 17.9% for anxiety and 11.6% for depression (35). The higher burden observed in our study may be attributed to the advanced disease stage and the administration of ICIs in later lines of therapy, as suggested in the literature (20, 21). Focusing more specifically on patients treated with ICIs, a recent review of eleven studies evaluating LTS treated with ICIs reported prevalence rates ranging from 30% to 82%, considerably higher than those seen in general cancer survivorship populations (9). Although the comparative data are limited, based on these observations and our cohort, we think that patients treated with ICIs could have at least the same risk level with patients treated with other anti-cancer treatments for psychosocial issues and probably a higher risk compared to normative cancer patient population.

While mood symptoms have been relatively well explored in ICI-treated survivors, FoP remains an underrecognized yet clinically significant dimension of psychological distress, affecting nearly half of our cohort (49.3%). Thewes et al. reported that 62% of adolescent and young adult cancer survivors experienced high levels of FoP (36), a proportion notably higher than typically reported rates in mixed adult cancer populations (37, 38). In our study, depression, anxiety and FoP were associated with a lower score in all the EORTC QLQ-C30 subscales. These findings highlight the importance of routine screening and timely management of psychological concerns in the follow-up care of ICI-treated cancer survivors, as addressing these issues may contribute to improved QoL.

ICIs are frequently accompanied by irAEs, which can range from mild to severe and affect various organ systems. In our cohort, over half of the patients experienced at least one irAE, with endocrine and cutaneous toxicities being the most common, and 8.4% developing grade ≥3 irAEs. Despite their frequency, irAEs were not significantly associated with impaired QoL, consistent with previous studies suggesting that chronic toxicity does not necessarily predict lower QoL among LTS (23, 33, 39). For example, Candido et al. found no association between irAEs and QoL deterioration two years after ICI initiation, indicating that psychological and functional factors may play a more dominant role in long-term well-being (23). Similarly, Özkan et al. reported that severe irAEs were not independently associated with worse QoL or physical functioning in older adults, even among frail individuals (39). Notably, irAEs were more common in patients receiving combination ICI regimens, particularly nivolumab-ipilimumab, yet these patients did not report disproportionately lower functional scores in our LTS cohort. In addition to self-reported QoL, RTW outcomes provided objective insight into functional recovery. Among the 173 patients who were employed at ICI initiation, only 88 (25.4%) were still working at the time of study visit, corresponding to a RTW rate of approximately 51%. Notably, over one-third of those who returned to work did so within the first six months. To best our knowledge, this data represent the largest body of evidence on RTW in ICI-treated patients with cancer, and suggest that despite long-term toxicity and advanced-stage disease, a significant proportion of LTS achieve occupational reintegration. Similar RTW rates have been reported in recent ICI-treated cohorts, including those receiving neoadjuvant immunotherapy, where shorter treatment duration and lower chronic toxicity could contribute to improved work resumption (40, 41). These findings suggest that treatment-related factors, including the length and intensity of ICI exposure, may meaningfully influence work participation outcomes in survivorship. They also highlight the importance of incorporating functional endpoints, such as return to work, into future immunotherapy survivorship research to better understand real-world recovery patterns. However, it should be noted that even in the adjuvant setting up to 40% of the patients did not resume occupation, highlighting the need for multi-stakeholder collaboration for job safety for ICI-treated LTS.

Our study has several limitations. First, the cross-sectional design precludes causal inference and does not allow for assessment of temporal changes in QoL or psychological symptoms and the absence of randomization together with multicenter recruitment may introduce selection bias and institutional heterogeneity that could influence the observed associations. Longitudinal studies with baseline and follow-up measurements would better elucidate the trajectory of survivorship outcomes over time. Second, our reliance on self-reported questionnaires may introduce recall and social desirability bias. Third, although psychological measures were comprehensively captured, we did not include objective neurocognitive testing, sexual health assessments, or caregiver-reported outcomes, all of which are clinically meaningful dimensions in ICI survivorship and may influence overall well-being. In addition, although RTW was documented, the study was not able to examine factors that may influence the ability to resume employment, such as the nature of job responsibilities, the level of workplace support, socioeconomic conditions, or the presence of cancer related fatigue. Despite these limitations, our study provides valuable insights into the real-world survivorship experience of patients treated with ICIs. To our knowledge, this is one of the largest studies to comprehensively assess QoL, emotional burden, and functional outcomes among ICI-treated LTS.

## 5 Conclusion

This nationwide, multicenter study highlights significant survivorship challenges among LTS receiving ICIs. Despite overall QoL comparable to the general population, approximately one-third experienced poor QoL, mainly driven by psychological distress, including anxiety, depression, and FoP. Our findings emphasize the critical need for structured survivorship programs focusing on psychological support and functional rehabilitation in this expanding patient population.

## Data Availability

The original contributions presented in the study are included in the article/**Supplementary Material**. Further inquiries can be directed to the corresponding author.
