# Supplementary material for: Health-related quality of life and psychosocial outcomes in long-term survivors treated with immune checkpoint inhibitors: a nationwide multicenter study
Source: Front Immunol. 2025 Dec 10;16:1693295. doi: 10.3389/fimmu.2025.1693295 (PMC12727895; doi:10.3389/fimmu.2025.1693295)
Supplement: Supplementary file 1 [file Table1.docx]

**Supplementary Table 1.** Distribution of EORTC QLQ-C30 subscale scores, n (%)

| **Sub-scale** | **0-10** | **11-20** | **21-30** | **31-40** | **41-50** | **51-60** | **61-70** | **71-80** | **81-90** | **91-100** |
| --- | --- | --- | --- | --- | --- | --- | --- | --- | --- | --- |
| **Global Health Status** | 3 (0.9%) | 7 (2.0%) | 10 (2.9%) | 19 (5.5%) | 64 (18.5%) | 24 (6.9%) | 79 (22.8%) | 24 (6.9%) | 62 (17.9%) | 54 (15.6%) |
| **Physical Functioning** | 1 (0.3%) | 7 (2.0%) | 10 (2.9%) | 25 (7.2%) | 13 (3.8%) | 53 (15.3%) | 36 (10.4%) | 80 (23.1%) | 46 (13.3%) | 75 (21.7%) |
| **Role Functioning** | 5 (1.4%) | 2 (0.6%) | 0 (0.0%) | 20 (5.8%) | 32 (9.2%) | 0 (0.0%) | 77 (22.3%) | 0 (0.0%) | 47 (13.6%) | 163 (47.1%) |
| **Emotional Functioning** | 3 (0.9%) | 1 (0.3%) | 4 (1.2%) | 6 (1.7%) | 28 (8.1%) | 24 (6.9%) | 55 (15.9%) | 52 (15.0%) | 48 (13.9%) | 125 (36.1%) |
| **Cognitive Functioning** | 3 (0.9%) | 5 (1.4%) | 0 (0.0%) | 14 (4.0%) | 28 (8.1%) | 0 (0.0%) | 67 (19.4%) | 0 (0.0%) | 107 (30.9%) | 122 (35.3%) |
| **Social Functioniing** | 9 (2.6%) | 4 (1.2%) | 0 (0.0%) | 20 (5.8%) | 32 (9.2%) | 0 (0.0%) | 98 (28.3%) | 0 (0.0%) | 59 (17.1%) | 124 (35.8%) |
